# Modified Corn Straw Hydrogel Adsorbent Fabricated via Embedding for Removing Cu^2+^ and Pb^2+^ from Wastewater

**DOI:** 10.3390/polym18212653

**Published:** 2026-10-26

**Authors:** Meiqing Fan, Hong Zhang, Wenming Wu, Hongze An, Xin Zhang, Xu Zeng, Xiaodong Yang, Bo Ren, Bo Wang

**Affiliations:** 1 Jilin Provincial Key Laboratory of Straw-Based Functional Materials, Institute for Interdisciplinary Biomass Functional Materials Studies, Jilin Engineering Normal University, Changchun 130052, China; 2 School of Biological and Food Engineering, Jilin Engineering Normal University, Changchun 130052, China; 3 Zhongxin Link Environmental Technology (Anhui) Co., Ltd., Lu’an 237000, China

**Keywords:** corn straw hydrogel adsorbent, carboxyl modification, embedding method, heavy metal adsorption

## Abstract

In this study, a modified corn straw hydrogel adsorbent (CSHA) was fabricated via the embedding method. The adsorbent facilitates phase separation after adsorbing lead ions (Pb^2+^) and copper ions (Cu^2+^) from wastewater. A set of experiments verified that metal ions formed coordination bonds with the carboxyl functional groups on the adsorbent surface. The influences of pH, contact time, initial metal ion concentration and temperature on adsorption performance were systematically explored. The results revealed that the adsorption capacities for Cu^2+^ and Pb^2+^ were 396.43 mg/g and 151.96 mg/g, respectively, under the given experimental conditions. The adsorption process followed the pseudo-second-order kinetic model and the Langmuir isotherm model. The thermodynamic calculations indicated that the adsorption was an endothermic and spontaneous process accompanied by increased system disorder. After five adsorption–desorption cycles, the removal efficiency of CSHA remained above 65%. This work offers a low-cost, eco-friendly and high-performance adsorbent for heavy-metal-containing wastewater treatment.

## 1. Introduction

Heavy metal pollution is one of the most serious environmental problems. With the acceleration of industrialization, the discharge of heavy-metal-containing wastewater from industries such as electroplating, metallurgy, tanning, and mining has increased year by year. Among these, lead (Pb^2+^) and copper (Cu^2+^) are the two most common heavy metal pollutants [1]. Lead is highly neurotoxic, causing irreversible damage to the human nervous system, hematopoietic system, and kidneys even at low concentrations, with particularly profound effects on children’s intellectual development [2,3]. Although copper is an essential trace element for the human body, excessive intake can lead to liver damage, gastrointestinal disorders, and renal tubular injury [4,5]. Due to their non-biodegradability, persistence, and bioaccumulation, heavy metal ions continue to exist in aquatic environments and are transmitted through the food chain, ultimately threatening the safety of ecosystems and human health. Therefore, the development of efficient, economical, and environmentally friendly technologies for treating heavy metal wastewater has become a research hotspot in the field of environmental science.

At present, the mainstream technologies employed for the disposal of heavy-metal-containing wastewater involve chemical precipitation, ion exchange, membrane separation, electrochemical treatment, and adsorption [6,7,8]. However, these conventional methods suffer from distinct drawbacks: chemical precipitation generates massive amounts of sludge waste; ion exchange encounters separation challenges, and regeneration expenses are high; membrane separation features intensive energy consumption and stringent influent quality criteria; while electrochemical treatment experiences severe electrode attrition and uses excessive energy [9,10,11]. In contrast, adsorption has been recognized as one of the most promising strategies owing to its facile operation, cost efficiency, exceptional removal efficacy, extensive applicability, and freedom from secondary contamination. The core determinant of adsorption performance is the screening of adsorbent materials. Despite the superior adsorption capacity of traditional adsorbents represented by activated carbon, their prohibitive preparation and regeneration costs impede their large-scale engineering application. Accordingly, exploiting novel adsorbents that are low cost and renewable with abundant reserves has become a pivotal research direction in this field.

As typical agricultural and forestry waste, agricultural straw has significant advantages such as wide availability, large output, low cost, and biodegradability. According to statistics, the annual output of crop straw in China exceeds 800 million tons, of which corn straw accounts for a considerable proportion [12,13]. Cellulose, hemicellulose and lignin constitute the major components of corn straw, whose molecular chains contain many active functional groups such as hydroxyl, carboxyl and amino groups. These functional groups can bind with heavy metal ions through complexation or electrostatic attraction, providing the theoretical basis for its use as an adsorbent [14,15]. However, when used directly as an adsorbent, raw corn straw has limitations, such as low adsorption capacity for heavy metals, poor selectivity, and insufficient mechanical strength. Therefore, appropriate modification of raw corn straw is necessary to improve its adsorption performance and stability.

Research on the modification of corn straw has made progress. Commonly used modification methods include physical modification (such as pyrolysis to produce biochar), chemical modification (such as acid/alkali treatment, graft copolymerization, and surface chelation functionalization), and biological modification [16,17,18]. Chemical graft modification is a mild and efficient approach for the chemical modification of biomass. Unlike modification methods such as high-temperature pyrolysis or strong acid/alkali oxidation, the reaction conditions of this method are controllable, theprocess is simple, and handling of by-products is easy. For example, Liu et al. synthesized a new type of adsorbent, mercaptoacetyl corn straw (MACS), by using the acid treatment method. When the adsorbent dosage was 20 g∙L^−1^, the adsorption temperature was 298 K, the initial pH was 6.0, the oscillation rate was 250 rpm, and the adsorption time was 40 min, and the maximum removal efficiency of Cu(II) was 93.57% [19]. Three biochar materials were produced from corn straw by Zeng et al. through individual modification with phosphoric acid, sodium hydroxide and zinc chloride for the adsorption of the first-generation oral antibiotic cephalexin. The results revealed that zinc-chloride-modified biochar contained more mesopores, with a specific surface area as high as 1494.9 m^2^∙g^−1^. The adsorption isotherm conformed to the Langmuir model, and the maximum adsorption capacity was 230.88 mg∙g^−1^ [20]. Wang et al. used corn straw as a raw biomass material, and prepared modified activated mesoporous carbon with citric acid, ZnCl_2_, NaOH or (NHH_4_)_2_Fe(SOO_4_)_2_. The Cr(VI) adsorption performance of different adsorbents was investigated. Among these adsorbents, the adsorbent modified with ZnCl_2_ exhibited an microporous structure, and its Cr(VI) removal rate reached 99.6 wt% [21].

In this work, corn straw was used as the raw material, and carboxyl-modified corn straw was prepared via chemical grafting. To overcome the poor separability of powdered biomass from aqueous solution, the modified corn straw was further fabricated into a composite hydrogel by an embedding method, yielding an adsorbent with high stability, that was easy separation, and had improved selectivity. Although various modification strategies for corn straw have been reported, most studies have focused on single physical or chemical modification, and few have systematically combined carboxyl functionalization with hydrogel immobilization. Compared with conventional lignocellulose-based hydrogels or recently reported corn straw adsorbents, the proposed approach avoids excessive structural damage to biomass under harsh conditions and achieves synchronous improvements in adsorption capacity, mechanical stability, and solid–liquid separation performance. By investigating the adsorption kinetics, isotherms, and effects of solution pH, the adsorption mechanism toward Pb^2+^ and Cu^2+^ was systematically revealed. This study not only highlights the advantages of integrating carboxyl modification and hydrogel embedding but also addresses the gaps in selectivity, reusability, and separation efficiency that exist in previous biomass hydrogel systems. This work provides a feasible route for the high-value utilization of corn straw and offers theoretical support for the efficient purification of heavy-metal-contaminated wastewater.

## 2. Materials and Methods

### 2.1. Synthesis

#### 2.1.1. Preparation of Carboxyl-Modified Corn Stalk (CCS)

A total of 2 g of 100-mesh corn stalk powder, 1 g of disodium hydrogen phosphate, 0.4 g of citric acid were added to 100 mL water. After stirring for 30 min, the mixture was poured into a glass dish and reacted at 55 °C for 24 h. The temperature was then raised to 100 °C for 100 min. After the reaction, the product was washed until the pH was 7. The obtained product was the carboxyl-modified corn stalk. The sample was marked CCS. For comparison, the unmodified corn stalk was labeled CS.

#### 2.1.2. Preparation of Corn Stalk Hydrogel Adsorbent (CSHA)

We added 10 mg of modified corn stalk, 50 mg of sodium alginate to 2 mL of water; after being mixed evenly, mixture A was obtained. Additionally, 3 g of calcium chloride was added to 80 mL of water, to obtain solution B. The aforementioned mixture A was dropped into solution B; the modified corn stalk water gel adsorbent was obtained after washing and drying. The sample was labeled CSHA. The adsorbent that adsorbed Cu^2+^ was labeled CSHA-Cu, and the adsorbent that adsorbed Pb^2+^ was labeled CSHA-Pb.

**Scheme 1:**
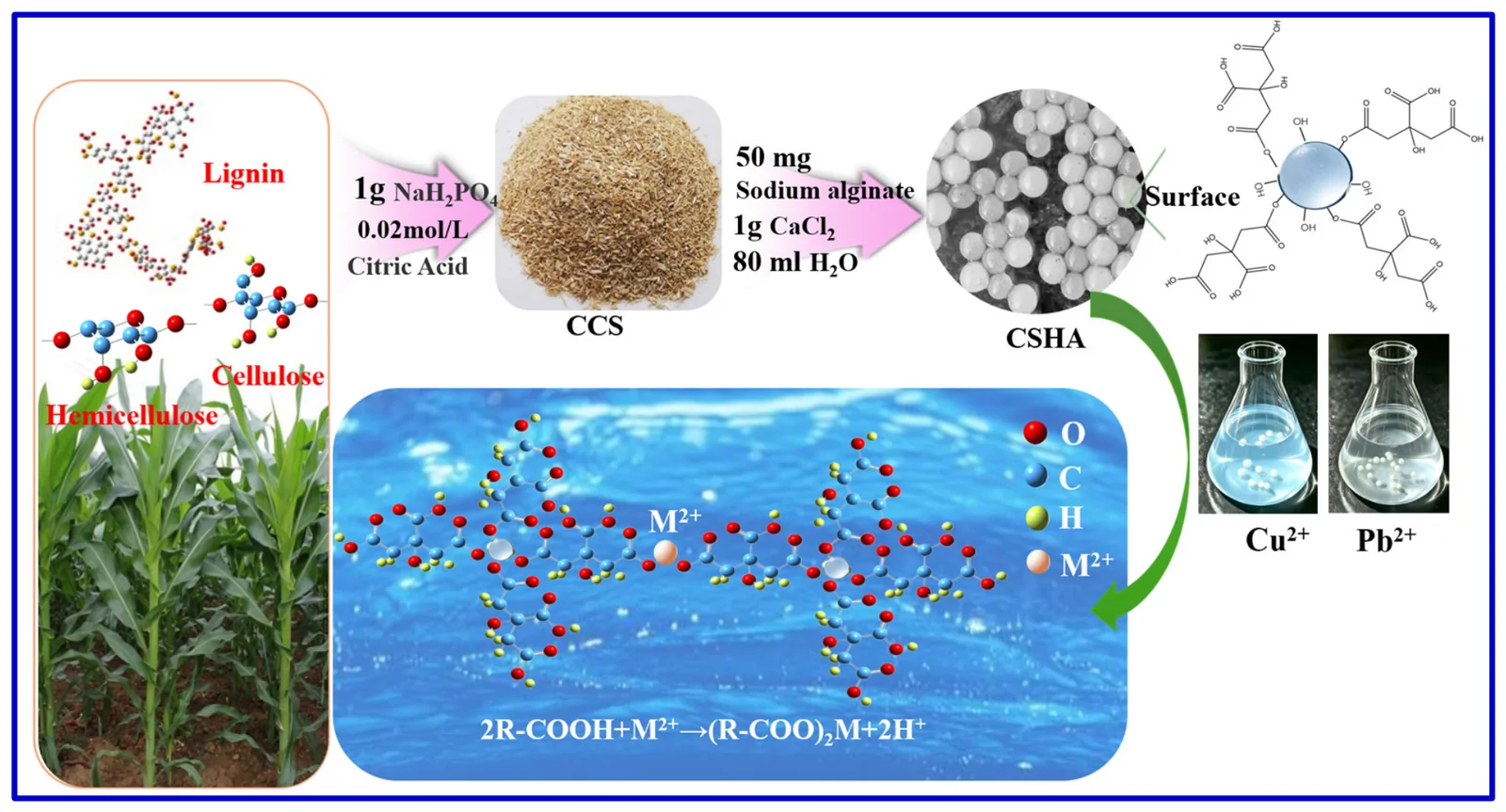
The possible mechanism of CSHA formation and adsorption process.

The possible mechanism of the CSHA formation and adsorption process is shown in Scheme 1.

### 2.2. Adsorption Experiments

#### 2.2.1. Effect of pH

A 100 mL solution was configured, in which both Cu^2+^ and Pb^2+^ possessed an initial concentration of 200 mg∙L^−1^. Then, 10 mg of adsorbent was added to the solution, followed by pH adjustment to 2–4 using 1 mol∙L^−1^ HCl and 1 mol∙L^−1^ NaOH. The solution was transferred to a thermostatic shaking device and agitated at 25 °C and 120 r∙min^−1^ for 160 min and 180 min separately to identify the optimal pH for Cu^2+^ and Pb^2+^ adsorption. We calculated the equilibrium adsorption amount using Formula (1) [22]:

(1) $$Q_{e}=\frac{(C_{0}-C_{e})V}{m}$$

where Q_e_ represents the adsorption equilibrium quantity, m is the amount of adsorbent, V is the volume of the solution, C_0_ is the initial concentration of the heavy metal ions, and C_e_ is the residual concentration of the heavy metal ions in the solution after adsorption.

#### 2.2.2. Effect of Contact Time

To explore the rate-limiting steps during adsorption, the pseudo-first-order kinetic model, pseudo-second-order kinetic model and intraparticle diffusion model were adopted to fit the adsorption data obtained at different reaction durations. The initial concentrations of Cu^2+^ and Pb^2+^ were both set at 200 mg∙L^−1^. After adding 10 mg of adsorbent, the solution pH was adjusted to 3. The mixture was transferred into a thermostatic shaking incubator and agitated at 25 °C with a rotation speed of 120 r∙min^−1^. The sampling time points were 20, 40, 60, 80, 100, 120, 140, 160, 180, 200 and 220 min.

#### 2.2.3. Effect of Heavy Metal Concentration

To further explore the adsorption mechanism, the Langmuir and Freundlich models were employed to fit the adsorption data. The initial concentrations of Cu^2+^ and Pb^2+^ were 50 mg∙L^−1^, 100 mg∙L^−1^, 150 mg∙L^−1^, 200 mg∙L^−1^, 250 mg∙L^−1^, and 300 mg∙L^−1^. Then, 10 mg of adsorbent, at pH 3, was placed in a constant-temperature shaking box and shaken at 25 °C at a rotational speed of 120 r∙min^−1^ for 160 min or 180 min. The optimal initial heavy metal concentration was investigated.

#### 2.2.4. Effect of Temperature

To further investigate the thermodynamic properties of the adsorbent, the initial concentrations of Cu^2+^ and Pb^2+^ were both 200 mg∙L^−1^; 10 mg of adsorbent, with pH 3, was placed in a constant-temperature shaking box. It was shaken at 25 °C, 45 °C, 65 °C, 85 °C at a rotational speed of 120 r∙min^−1^, and the adsorption time was 160 min and 180 min.

### 2.3. Materials Characterization

The samples’ morphological features were characterized using scanning electron microscopy (SEM, JEOL JSM-IT810, JEOL Ltd., Tokyo, Japan). Functional group variations of the adsorbent upon modification were recorded with a Fourier-transform infrared spectrometer (FTIR, Avatar 370, Nicolet, Thermo Fisher Scientific, Madison, WI, USA). Heavy metal ion levels in the original and post-adsorption solutions were determined by inductively coupled plasma mass spectrometry (ICP-MS, 820-MS, Bruker, Billerica, MA, USA). The chemical constituents of CSHA, CSHA-Cu and CSHA-Pb were further investigated by X-ray photoelectron spectroscopy (XPS, Thermo Scientific ESCALAB 250 XI).

## 3. Results and Discussion

### 3.1. Material Properties

**Figure 1:**
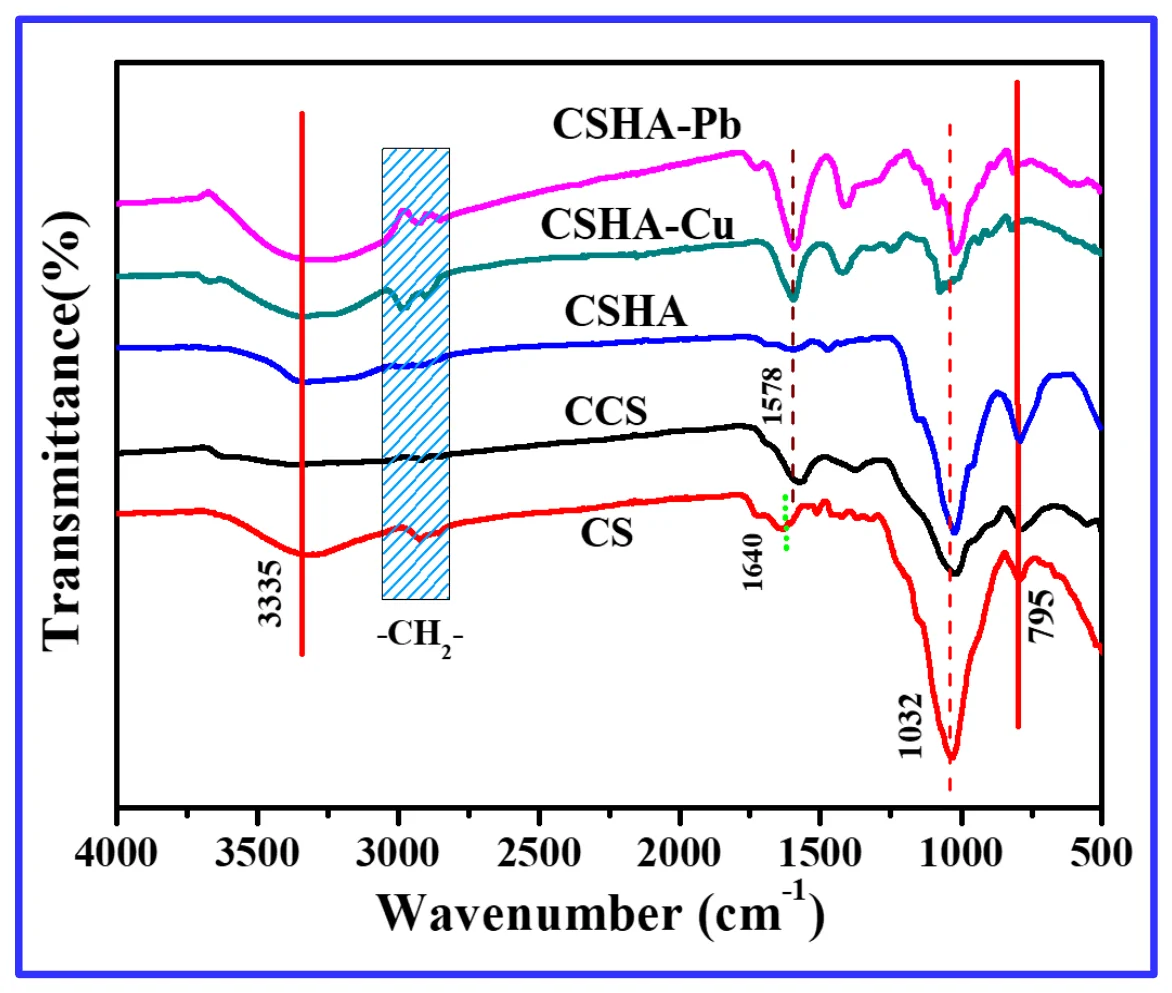
FTIR spectra of different samples.

Figure 1 presents the FTIR spectra of corn stalk (CS), modified corn stalk (CCS), corn stalk hydrogel adsorbent (CSHA), CSHA-Cu and CSHA-Pb. The broad band centered at 3335 cm^−1^ corresponds to the O-H stretching vibration of hydroxyl groups and adsorbed water molecules. The absorption band around 2900 cm^−1^ was assigned to the C-H stretching vibration of -CH_2_- groups originating from the alkyl skeleton of cornstalk polysaccharides [23]. For CS, the peak at 1640 cm^−1^ belonged to C=O stretching of protonated carboxyl groups. After chemical modification, the characteristic peak shifted from 1640 cm^−1^ to 1578 cm^−1^, which was attributed to the formation of deprotonated carboxylate (-COO^−^) [24]. The generation of carboxylate groups confirmed successful modification, and abundant active sites were introduced onto the cornstalk matrix. The intensive peak at 1032 cm^−1^ and the weak band at 795 cm^−1^ were attributed to C-O/C-O-C stretching and sugarring vibration, respectively, demonstrating that the basic polysaccharide skeleton was well-preserved after modification [25]. For CSHA, the peak at 3335 cm^−1^ further broadened, indicating that there were large numbers of hydroxyl groups in the composite hydrogel, and the C-O peak at 1032 cm^−1^ was retained with enhanced intensity. This proved that sodium alginate was successfully introduced, and the polysaccharide framework was intact [26,27,28]. After Cu^2+^ and Pb^2+^ adsorption, no new characteristic peaks of metal–oxygen bonds were observed, because M-O stretching vibrations mainly appeared in the far-infrared region below 400 cm^−1^, which was not covered in this measurement range. Nevertheless, obvious shifts and profile changes were observed for -OH (3335 cm^−1^), -COO^−^ (1578 cm^−1^), and C-O (1032 cm^−1^), verifying that hydroxyl and carboxylate groups took part in chelating interactions with Cu^2+^ and Pb^2+^. Different degrees of peak shifts between CSHA-Cu and CSHA-Pb reflected the difference in coordination strength between the two metal ions and oxygen-containing sites, which is consistent with the XPS results and HSAB theory.

**Figure 2:**
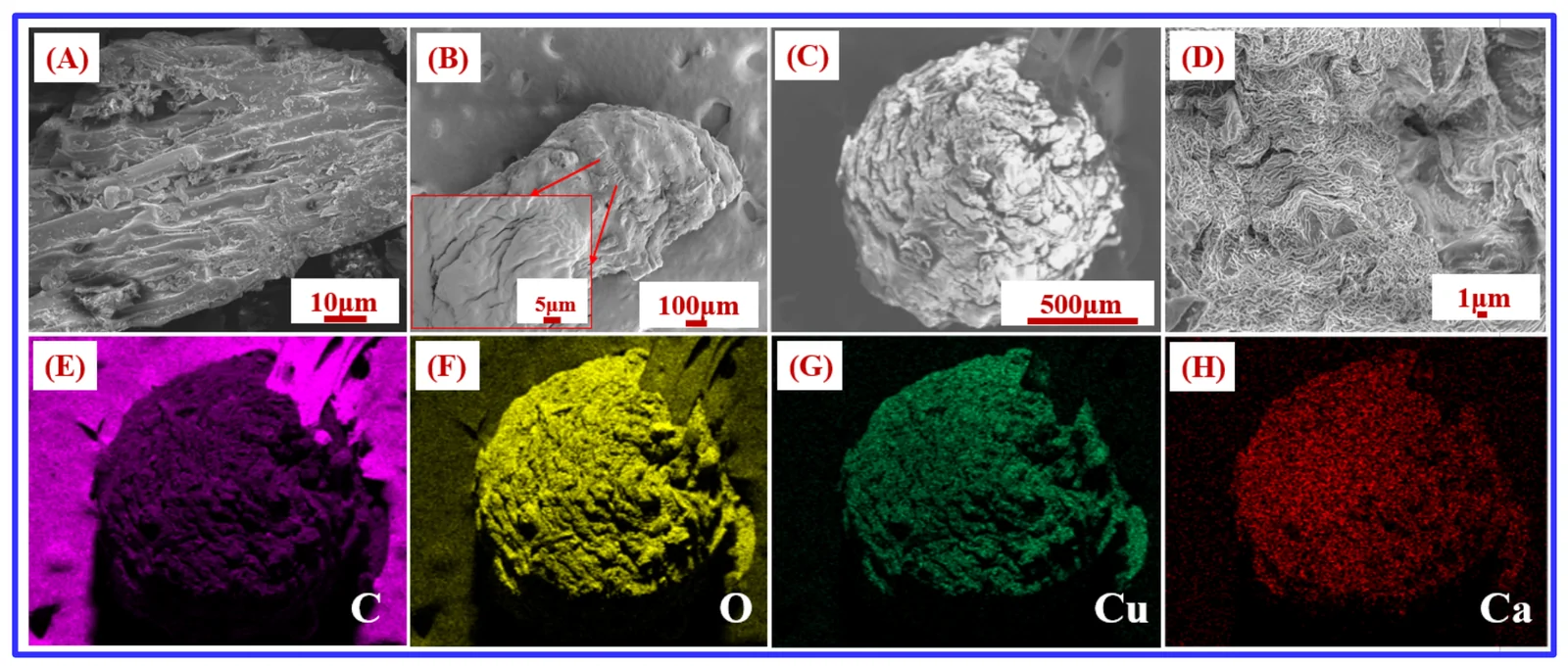
SEM of modified corn stalks (**A**); SEM of CSHA (inset is magnified SEM image) (**B**); low-magnification SEM image (**C**) and high-magnification SEM image (**D**) of CSHA-Cu and corresponding elemental mapping images (**E**–**H**).

**Figure 3:**
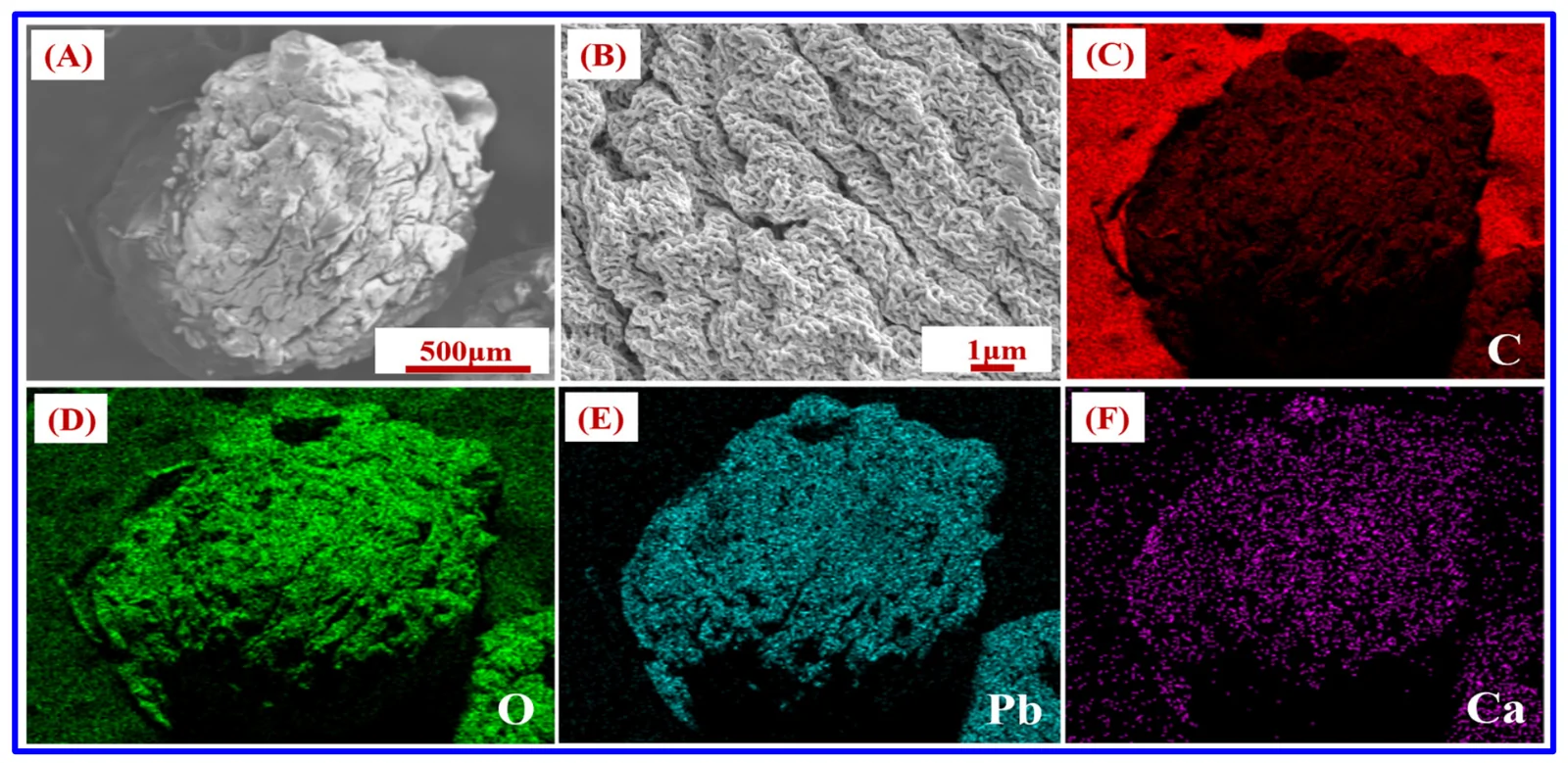
Low-magnification SEM image (**A**) and high-magnification SEM image (**B**) of CSHA-Pb and corresponding elemental mapping images (**C**–**F**).

Figure 2A shows the SEM image of the modified corn stalk. As can be seen, the modified corn stalk retained the natural plant fiber structure, with a rough and uneven surface, and contained large numbers of grooves and pores. This uneven fiber structure could provide sufficient bonding sites for the construction of the hydrogel. Figure 2B shows the SEM image of the CHSA. As can be seen from the figure, the adsorbent had fewer surface wrinkles than after adsorbing heavy metal ions. This is because one heavy metal ion can simultaneously coordinate with the carboxyl groups on two polymer chains, resulting in secondary cross-linking, polymer network tightening, and overall swelling and dehydration, and the surface and internal contraction are not synchronized, resulting in more wrinkles [29,30]. Low-magnification SEM (Figure 2C and Figure 3A) shows that the hydrogel spheres retained intact spherical morphology after metal-ion adsorption; numerous folds appeared on the sphere surface, which resulted from the contraction of the hydrogel internal structure during adsorption. High-magnification SEM (Figure 2D and Figure 3B) was used to further observe the microscopic morphology. After adsorption, the surface of the material was uneven, and there were abundant pore structures. These structures can provide adsorption channels and active sites for metal ions, facilitating the diffusion of metal ions into the interior of the adsorbent. Figure 2E–H and Figure 3C–F show the elemental mapping images of the hydrogel microspheres after adsorption. From the figures, it can be seen that C and O were uniformly distributed throughout the entire microsphere area, representing the skeleton elements of the hydrogel organic matrix, which proved that the corn-straw-based hydrogel matrix was intact; Cu (Figure 2G) and Pb (Figure 3E) were uniformly distributed throughout the entire microsphere range, indicating that metal ions not only adsorbed on the surface of the adsorbent but also successfully diffused into the interior of the adsorbent; Ca (Figure 2H and Figure 3F) was also evenly distributed throughout the sphere, and Ca originated from the cross-linking components in the hydrogel preparation process. Based on the combined morphology and elemental distribution results, it was found that this modified corn straw-based hydrogel adsorbent effectively captures Cu^2+^ and Pb^2+^.

**Figure 4:**
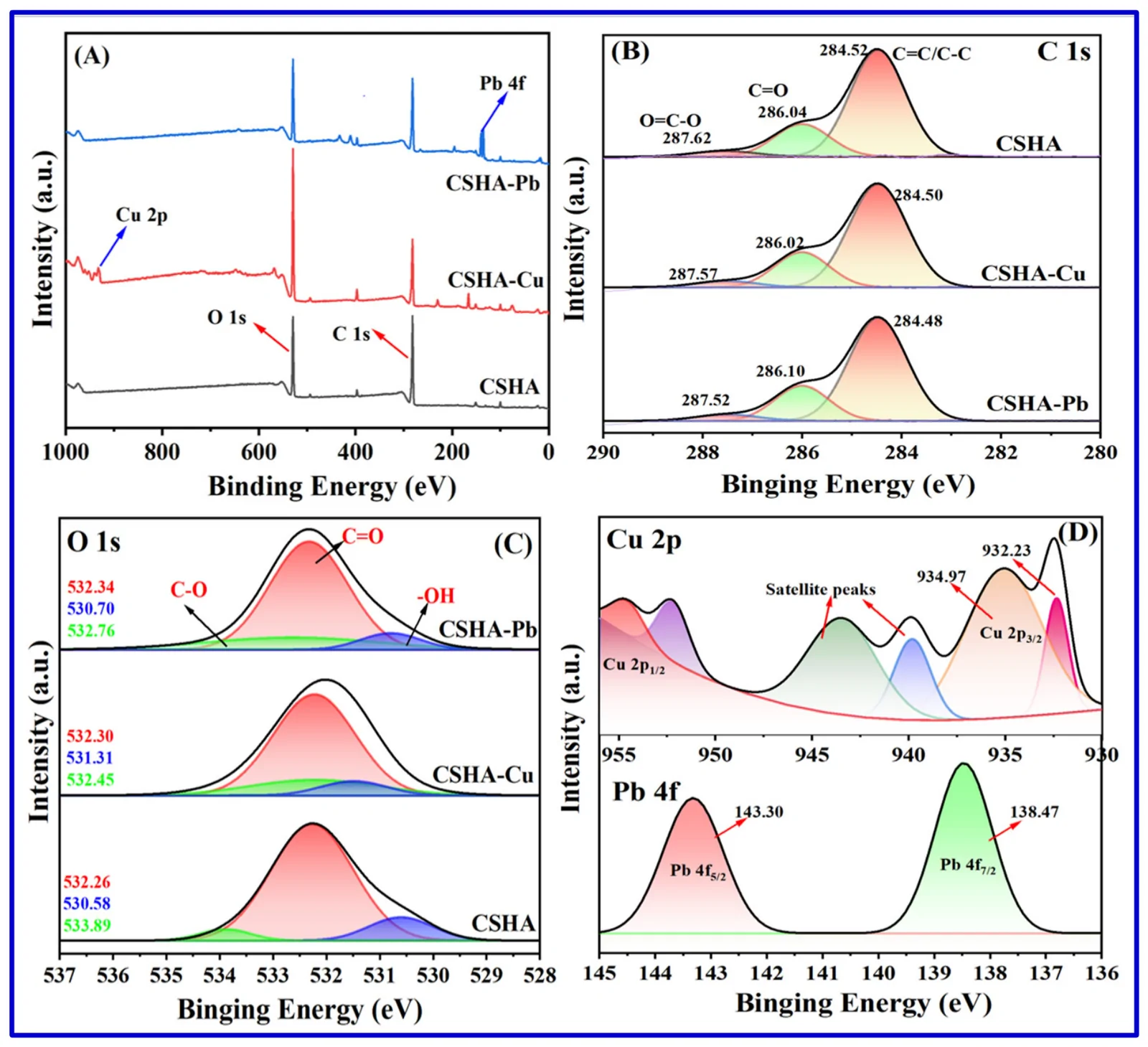
(**A**) Total XPS spectrum of different samples; (**B**) C 1s XPS spectrum of different samples; (**C**) O 1s XPS spectrum of different samples; (**D**) Cu 2p and Pb 4f XPS spectra of CSHA-Cu and CSHA-Pb, respectively.

As shown in Figure 4A, compared with CSHA, CSHA-Cu and CSHA-Pb exhibited two new Pb 4f and Cu 2p peaks, indicating that Cu^2+^ and Pb^2+^ adsorbed on CSHA. As can be seen from Figure 4B, after the adsorption of Cu^2+^ and Pb^2+^, the area ratio of the C=O bond peak decreased, indicating that the oxygen-containing functional groups participated in the adsorption process [31,32,33,34]. As can be seen from Figure 4C, after the adsorption of Cu^2+^ and Pb^2+^, the relative peak area of -OH significantly decreased, indicating that the hydroxyl group was consumed; -OH lost H^+^ and formed a coordination bond with the metal ion. The peak areas of C-O and C=O decreased significantly, indicating that the functional groups participated in chelation and were consumed. Figure 4D shows the XPS spectra of Cu 2p and Pb 4f. It can be seen that after adsorbing Cu^2+^, peaks of Cu 2p_1/2_ and Cu 2p_3/2_ appeared. After adsorbing Pb^2+^, peaks of Pb 4f_5/2_ and Pb 4f_7/2_ appeared at 143.30 eV and 138.47 eV respectively [35]. This indicated that Cu^2+^ and Pb^2+^ adsorbed on the surface of CSHA in the form of coordination bonds.

### 3.2. Adsorption Properties

#### 3.2.1. Effects of pH

**Figure 5:**
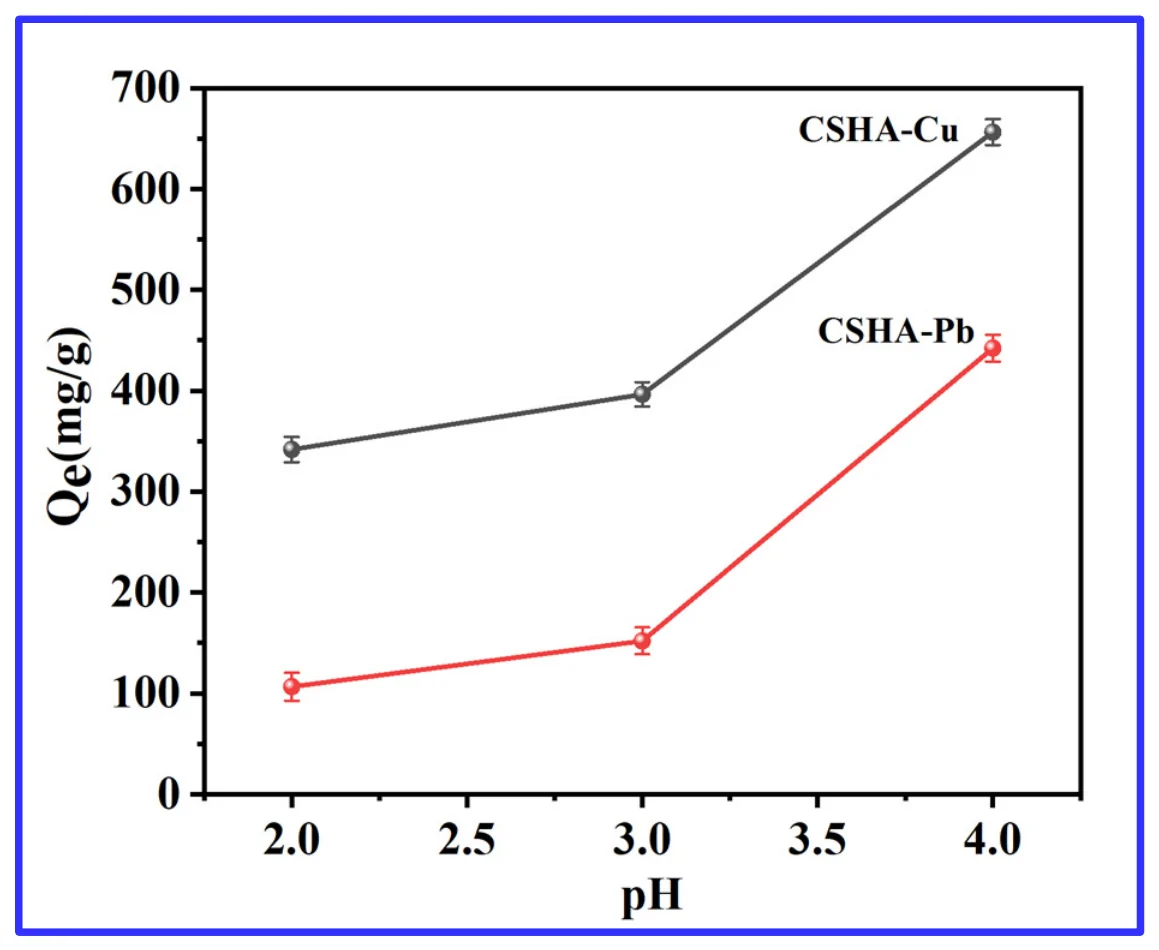
Effects of pH value.

Figure 5 illustrates the effect of varying the pH conditions on the adsorption performance of the adsorbent for metal ions. The adsorption capacity for both target metal cations improved remarkably with the elevation of solution pH. In the low-pH environments, abundant hydrogen ions existed in the aqueous solutions, which competed fiercely with metal ions for the limited active sites on the adsorbent’s surface. Meanwhile, the surface functional groups of the adsorbent were protonated under acidic conditions, generating electrostatic repulsion against positively charged metal cations and ultimately inhibiting the adsorption reaction. Therefore, the adsorption capacity was low. As the pH value increased, the competition of H^+^ weakened, and the functional groups de-protonated and became negatively charged, enhancing the adsorption of Cu^2+^ and Pb^2+^ through electrostatic attraction [36,37]. However, trace precipitation occurred at pH4; thus, the pH was fixed at 3 in the subsequent experiments.

#### 3.2.2. Adsorption Kinetics

**Figure 6:**
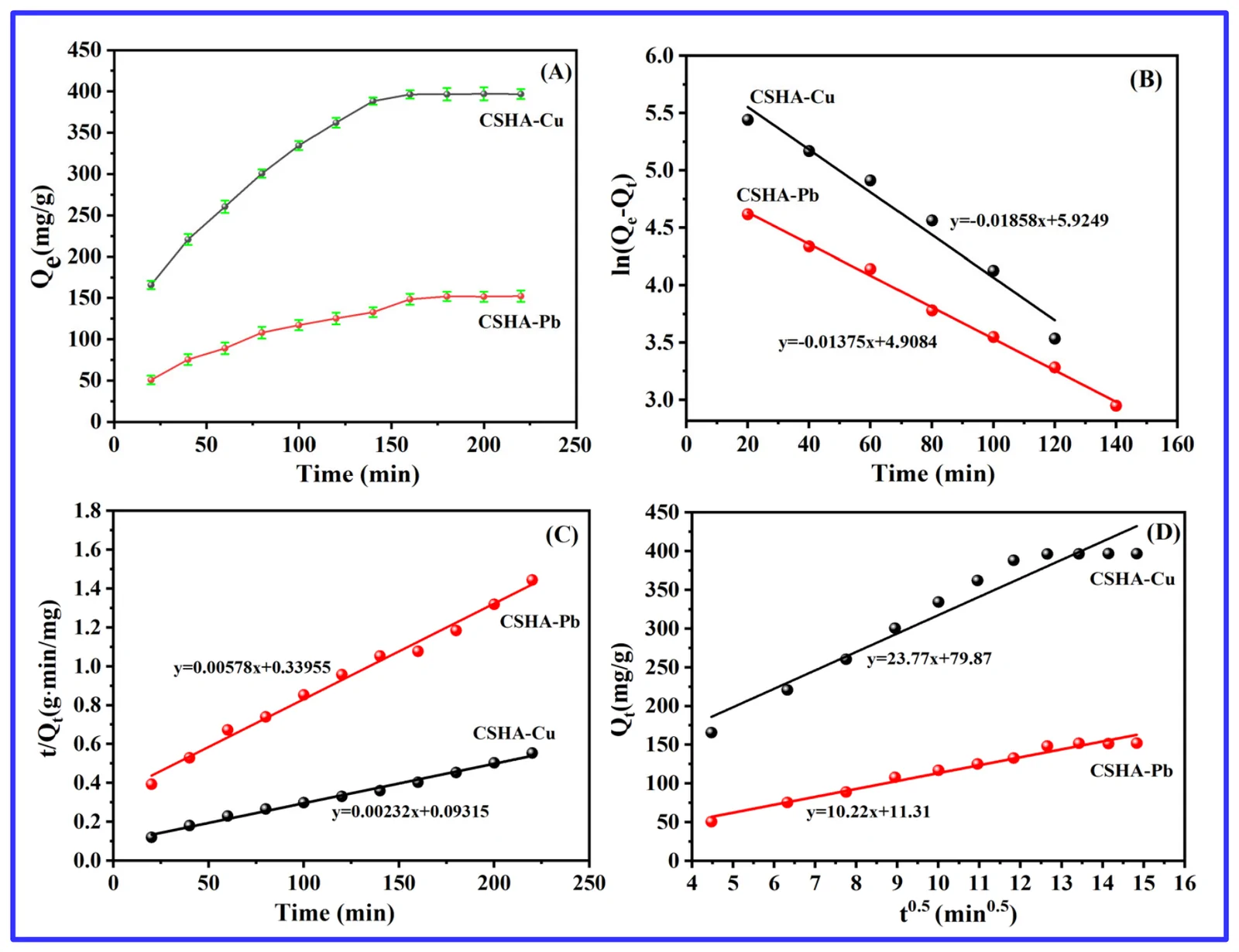
(**A**) Effects of contact time; (**B**) quasi-first-order kinetic model; (**C**) quasi-second-order kinetic model; (**D**) intra-particle diffusion model.

Figure 6A shows the effects of contact time. As shown in the figure, the adsorption rate rose rapidly in the initial reaction stage. This was because abundant free –COOH active sites existed on the adsorbent surface at the beginning, so the Cu^2+^ and Pb^2+^ in the solution rapidly diffused to the adsorbent surface and bound via coordination bonds. When the reaction time was 160 min and 180 min, the adsorption of Cu^2+^ and Pb^2+^ reached equilibrium respectively. The adsorption amounts were 396.43 mg∙g^−1^ and 151.96 mg∙g^−1^ respectively. The adsorption amount of Cu^2+^ being higher than that of Pb^2+^ could be attributed to the smaller radius of Cu^2+^ ions [38], which had lower diffusion resistance in the pores of the adsorbent and reached the internal adsorption sites more quickly.

Further analysis of the rate-limiting steps of this adsorption process was conducted using the pseudo-first-order kinetic model (Figure 6B), the pseudo-second-order kinetic model (Figure 6C), and the intragranular diffusion model (Figure 6D). According to Formulas (2)–(4), the linearly fitted data are shown in Table 1. As can be seen, the linear coefficient R^2^ of the pseudo-second-order kinetic model was closer to one, indicating that this adsorption process was controlled by quasi-second-order kinetics. According to FTIR and XPS analysis, it was also seen that chemical adsorption dominated the entire adsorption process. The carboxyl groups on the surface of the adsorbent underwent complexation reactions with Cu^2+^ and Pb^2+^ in the solution. The non-zero intercept X of the intra-particle diffusion model suggested that intra-particle-diffusion took part in the adsorption process, yet it was not the main rate-controlling step.

(2) $$\ln(Q_{e}-Q_{t})=\ln Q_{e}-\frac{K_{1}t}{2.303}$$

(3) $$\frac{t}{Q_{t}}=\frac{1}{K_{2}Q_{e}^{2}}+\frac{t}{Q_{e}}$$

*Q_t_* = *K*_id_t^0.5^ + *X* (4)

where t represents the adsorption time (min), Q_t_ represents the instantaneous adsorption amount (mg∙g^−1^), K_1_ is the coefficient of the pseudo-first-order adsorption rate (min^−1^), K_2_ is the coefficient of the pseudo-second-order adsorption rate (g·mg^−1^·min^−1^), K_id_ is the coefficient of the intra-granular adsorption rate (g·mg^−1^·min^−1/2^), and X is the coefficient of the intra-granular diffusion rate (mg·g^−1^·min^−1^) [39,40].

#### 3.2.3. Adsorption Isotherms

**Figure 7:**
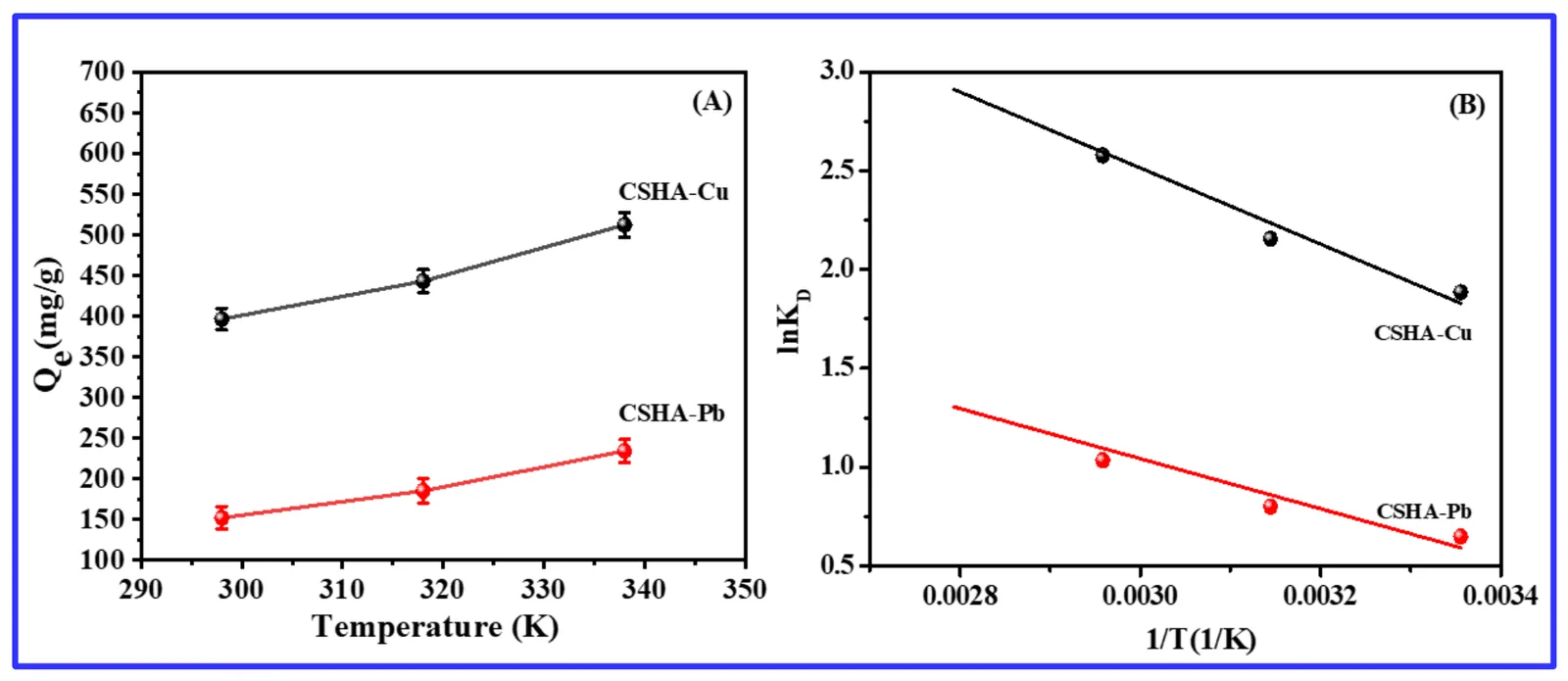
(**A**) Effects of temperature on adsorption capacity; (**B**) plots of 1/T vs. lnK_D_ for different samples.

As shown in Figure 7A, with the increase in temperature, the adsorption amounts of Cu^2+^ and Pb^2+^ gradually increased, indicating that the temperature rise was conducive to the forward adsorption process. This adsorption process was an endothermic process.

Figure 7B shows the fitting curve of lnK_D_ at different temperatures. By combining Formulas (5) and (6), the relevant thermodynamic parameters were obtained, as shown in Table 2. The calculated ΔH^0^ values were all greater than zero, indicating that both were endothermic reactions. ΔS^0^ was greater than zero, indicating that the disorder of the system increased after adsorption. With the increase in temperature, ΔG^0^ was always less than zero, indicating that the adsorption process was spontaneous. At the same temperature, the absolute value of CSHA-Cu was much greater than that of CSHA-Pb, indicating that the spontaneous degree and binding stability of adsorbing Cu^2+^ were much better than those of Pb^2+^, which is consistent with the comparison of the results of the kinetic rate.

(5) $$\ln K_{D}=-\frac{\Delta H^{0}}{\mathrm{RT}}+\frac{\Delta S^{0}}{R}$$

Δ*G*^0^=Δ*H*^0^ − *T*Δ*S*^0^ (6)

(7) $$K_{D}=\frac{Q_{e}}{C_{e}}=\frac{C_{0}-C_{e}}{C_{e}}\cdot \frac{V}{m}$$

where K_D_ is distribution coefficient (mL∙g^−1^); ΔH^0^ is enthalpy change (kJ∙mol^−1^); ΔS^0^ is entropy change (kJ∙mol^−1^∙K^−1^); ΔG^0^ is Gibbs free energy change (kJ∙mol^−1^) [41,42].

#### 3.2.4. Adsorption Mechanism

**Figure 8:**
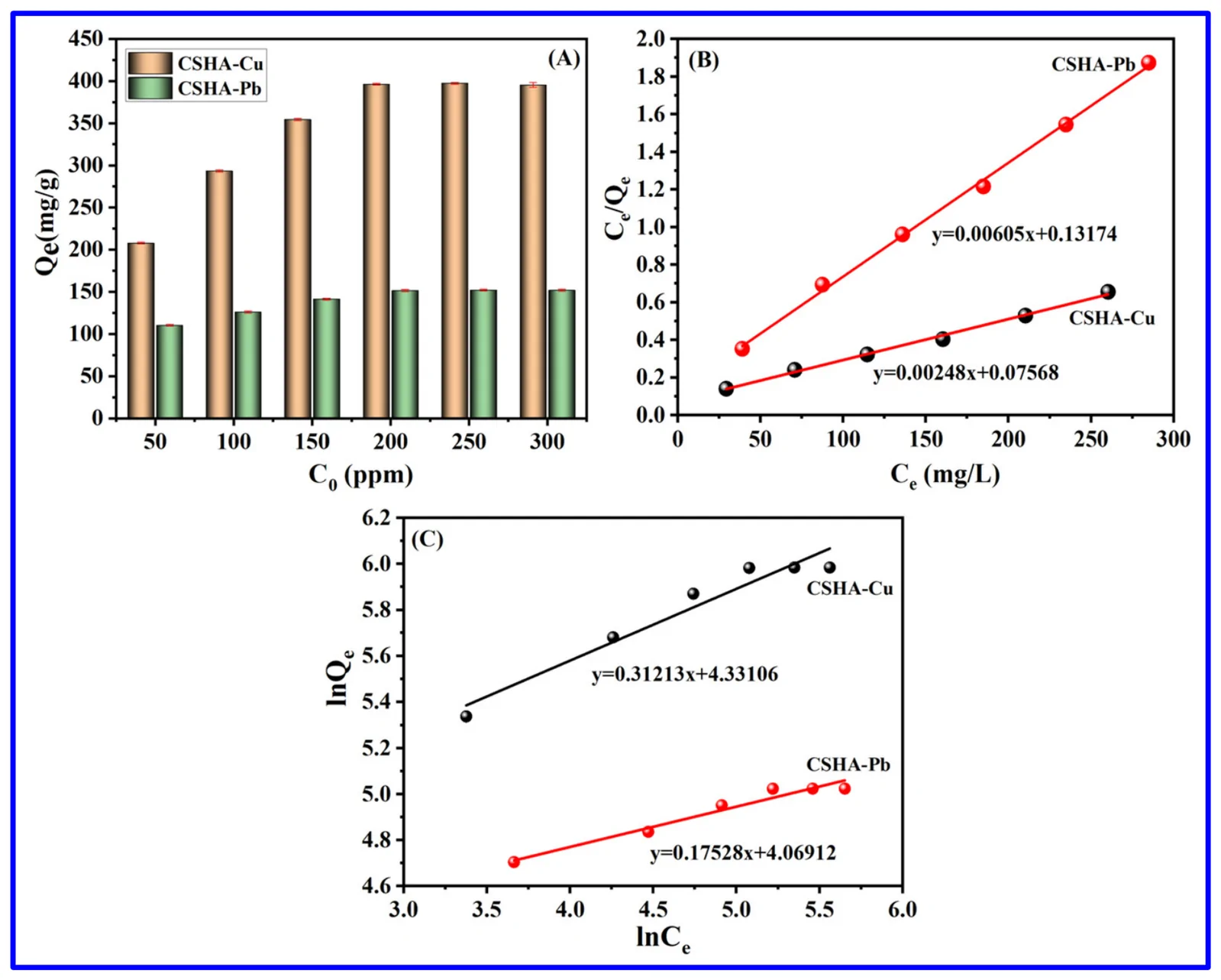
(**A**) Effects of initial heavy metal concentration; (**B**) Langmuir isothermal adsorption at different initial heavy metal concentrations; (**C**) Freundlich isothermal adsorption at initial heavy metal concentration.

Figure 8A shows a comparison of the adsorption amounts of Cu^2+^ and Pb^2+^ for different initial concentrations. It can be seen that the adsorption amounts of Cu^2+^ and Pb^2+^ gradually increased with increasing concentration. The reason for this can be attributed to the large numbers of -COOH and -OH active sites on the surface of the adsorbents at low concentrations. When the initial concentration exceeded 200 ppm, most active sites were occupied by metal ions, leaving very few vacancies. Further increasing the ion concentration would not significantly enhance the adsorption capacity. As can be seen from the figure, the equilibrium adsorption amount of Cu^2+^ was greater than that of Pb^2+^. The reasons for this can be summarized as follows:

Hard and soft acid–base (HSAB) theory can further explain the superior Cu^2+^ than Pb^2+^ adsorption capacity of CSHA.The oxygen-containing functional groups (-COOH, -OH and C=O) on CSHA act as hard bases, in which oxygen atoms serve as electron-donating sites. Cu^2+^ is classified as a borderline acid, while Pb^2+^ is a typical soft acid. According to the HSAB principles, borderline acids prefer to coordinate with hard bases, whereas soft acids have higher affinity for soft bases such as S- and N- containing groups. Therefore, the borderline acid Cu^2+^ exhibits stronger binding affinity toward the hardbase oxygen sites on CSHA than soft acid Pb^2+^, leading to the formation of more stable metal–oxygen chelating complexes. In addition due to the smaller ionic radius of Cu^2+^, Cu^2+^ can diffuse more easily into the internal pore structure of hydrogel microspheres and access more active sites. This synergistic effect of stronger coordination affinity (HSAB) and faster intraparticle diffusion jointly accounts for the higher adsorption capacity and better removal efficiency of Cu^2+^ compared with Pb^2+^.

Based on Formulas (8) and (9), the Langmuir (Figure 8B) and Freundlich (Figure 8C) adsorption isotherm fitting curves were plotted, and the calculated data are shown in Table 3. As can be seen, the R^2^ values of the Langmuir isothermal adsorption for both Cu^2+^ and Pb^2+^ ions were much closer to one, and the linear fitting was significantly better than that of the Freundlich isothermal adsorption. This indicated that both were single-layer adsorption [43,44]. The adsorption sites were uniform, and there was no interaction between the adsorbed molecules.

(8) $$\frac{C_{e}}{Q_{e}}=\frac{1}{K_{L}Q_{\max}}+\frac{C_{e}}{Q_{\max}}$$

(9) $$\ln Q_{e}=\ln K_{F}+\frac{\ln C_{e}}{n}$$

In the formulas, C_e_ represents the equilibrium concentration (mg∙L^−1^); Q_max_ is the maximum adsorption capacity (mg∙g^−1^); K_L_ is the coefficient of Langmuir adsorption; K_F_ and *n* are the coefficients of Freundlich adsorption [45,46].

#### 3.2.5. Comparison with Other Adsorbents

Table 4 presents a comparison of the adsorption properties of various adsorbents for Cu^2+^ and Pb^2+^ [47,48,49,50,51,52,53,54,55]. As can be seen from the table, the adsorbents developed in this work demonstrate significant advantages in terms of adsorption capacity and efficiency. Firstly, the maximum amounts of Cu^2+^ and Pb^2+^ adsorbed by the adsorbents in this work were 396.43 mg∙g^−1^ and 151.96 mg∙g^−1^ respectively, which are much higher than those of most of the adsorbents in the table. Furthermore, the adsorbents in this work simultaneously removed Cu^2+^ and Pb^2+^ within the applicable pH range. Many of the adsorbents in the table were tested only for a single metal ion, while our adsorbent can efficiently and simultaneously remove both heavy metals within a similar or broader pH range, demonstrating stronger practical wastewater treatment potential. In conclusion, the adsorbents prepared in this work outperform the other adsorbents listed in the table in terms of adsorption capacity, adsorption rate, and the ability to remove both metals simultaneously, showing obvious comprehensive performance advantages and application prospects.

#### 3.2.6. Recycle Performance

**Figure 9:**
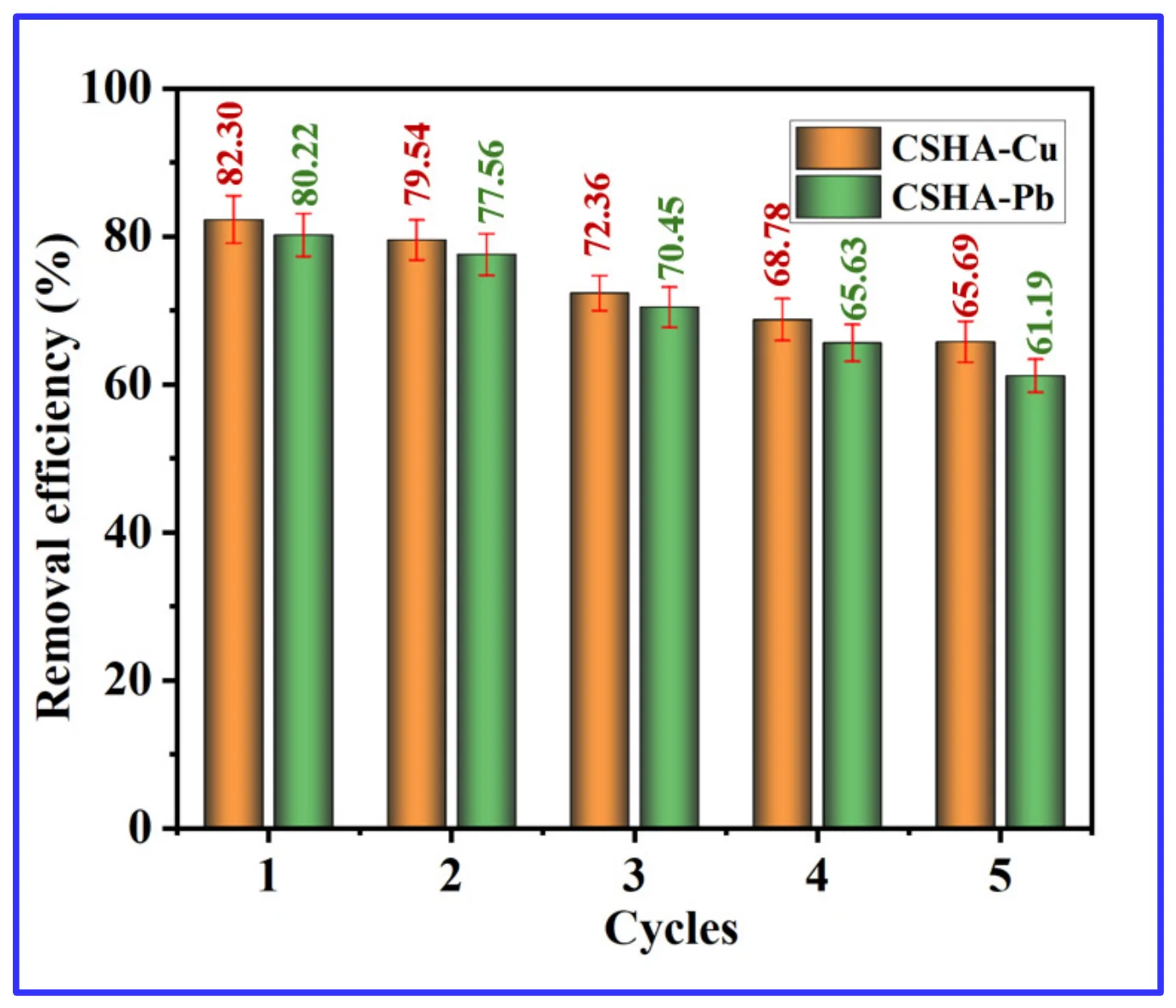
Recycle performance of adsorbent.

As shown in Figure 9, five adsorption–desorption cycles of Cu^2+^ and Pb^2+^ were carried out, with 0.1 M HCl used as the desorption agent. The results indicated that in the first cycle, the removal efficiencies for Cu^2+^ and Pb^2+^ reached 82.31 % and 80.22 %, respectively. After five cycles, they dropped to 65.78 % and 61.19 %. Thus, it can be seen that with the increase in the number of cycles, the removal efficiency showed a downward trend, which was attributed to the exhaustion of active adsorption sites and the incomplete desorption of Cu^2+^ and Pb^2+^.

## 4. Conclusions

In this work, a carboxyl-modified corn straw hydrogel adsorbent (CSHA) was prepared by the embedding method. The material characterization results demonstrated that the prepared CSHA possessed a rough porous surface and abundant internal pore structures, providing numerous active adsorption sites and mass transfer channels for heavy metal ions. FTIR analysis verified that abundant hydroxyl and carboxyl functional groups were successfully grafted onto the surface of the modified corn stalks, and the distinct shifts in the characteristic functional group peaks after metal ion adsorption confirmed the vital participation of oxygen-containing groups in the adsorption reaction. The combined XPS and elemental mapping results further revealed that oxygen atoms from hydroxyl, carbonyl and carboxyl groups and carbon atoms from the carbon skeleton of CSHA participated in the coordination chelation reaction with Cu^2+^ and Pb^2+^.

The batch adsorption experiments indicated that the as-synthesized adsorbent exhibited excellent adsorption performance toward Pb^2+^ and Cu^2+^. At pH = 3, equilibrium for Cu^2+^ and Pb^2+^ adsorption was established after 160 min and 180 min, with measured adsorption capacities of 396.43 mg∙g^−1^ and 151.96 mg∙g^−1^. Kinetic analysis showed that the pseudo-second-order kinetic model best described the adsorption process, demonstrating a predominance of chemisorption. The adsorption isotherm data were well-represented by the Langmuir model, pointing to a monolayer adsorption mechanism. The thermodynamic parameters demonstrated that the adsorption of both metal ions was an endothermic and spontaneous process, accompanied by an increase in system disorder. After five successive adsorption–desorption cycles, CSHA retained a removal efficiency above 65 %. In conclusion, the as-prepared corn-straw-based hydrogel adsorbent is a cost-effective and highly efficient material. Considering its low-cost raw material source, simple fabrication procedure and satisfactory reusability, CSHA shows promising application potential for practical heavy-metal-containing wastewater treatment.

## Figures and Tables

**Table 1:** Kinetic parameters for different samples.

Kinetic Models	Samples	K	Q_e,exp_ (mg∙g^−1^)	Q_e,cal_ (mg∙g^−1^)	R^2^
quasi-first-order	CSHA-Cu	0.043	396.43	374.24	0.9658
	CSHA-Pb	0.032	151.96	135.42	0.9862
quasi-second-order	CSHA-Cu	5.38	396.43	431.03	0.9918
	CSHA-Pb	3.34	151.96	173.01	0.9936
intra-particle diffusion		**K_id_** **(g·mg^−1^·min^−1/2^)**	**Q_e,exp_** **(mg∙g^−1^)**	**X** **(mg·g^−1^·min^−1^)**	**R^2^**
	CSHA-Cu	23.77	396.43	79.87	0.9339
	CSHA-Pb	10.22	151.96	11.31	0.9727

**Table 2:** Thermodynamic parameters of different samples.

Samples	ΔH^0^	ΔS^0^	ΔG^0^(kJ∙mol^−1^)		
	kJ∙mol^−1^	kJ∙mol^−1^∙K^−1^	298K	318 K	338 K
CSHA-Cu	16.010	0.069	−4.552	−5.932	−7.312
CSHA-Pb	10.538	0.040	−1.382	−2.182	−2.982

**Table 3:** Adsorption isotherm parameters for different samples.

Langmuir Isothermal Adsorption Model (298 K)				
Samples	Q_e,exp_ (mg∙g^−1^)	Q_max_ (mg∙g^−1^)	K_L_ (L∙mg^−1^)	R^2^
CSHA-Cu	396.43	403.23	30.52	0.99765
CSHA-Pb	151.96	165.29	21.72	0.99483
**Freundlich isothermal adsorption model (298 K)**				
**Samples**	**Q_e,exp_ (mg∙g^−1^)**	**K_F_ (L∙mg^−1^)**	***n***	**R^2^**
CSHA-Cu	396.43	76.02	3.20	0.93331
CSHA-Pb	151.96	54.50	5.71	0.94743

**Table 4:** Comparison of adsorption conditions and Cu^2+^ and Pb^2+^ adsorption capacity of different samples.

Adsorbents	Adsorption Conditions		Adsorption Capacity (mg∙g^−1^)		Refs.
	Contact Time (min)	pH	Cu^2+^	Pb^2+^	
*Hypotrachynacirrhata*	50	6	6.68	—	[47]
*Melia azedarach* leaves	120	5	48.63	48.51	[48]
*Melia azedarach* twigs			44.71	30.82	
Tobacco straw (ETS-PAA)	210	6	74.35	292.15	[49]
Bean husk/LDPE biochar	40	8	—	15.22	[50]
Bamboo fiber/chitosan (BF/CSA)	120	3	138.97	—	[51]
Dead cells of *Microcystis aeruginosa* blooms	90	6	—	75.19	[52]
Norway Spruce Wood Residue (NSWR)	20	5	7.9	10.3	[53]
Chitosan nanofibril	120	5	168.7	118	[54]
Sunflower stalks biochars	20	5	253.7	—	[55]
Corn stalk (CSHA)	160/180	3	396.43	151.96	This work

## Data Availability

The original contributions presented in this study are included in thearticle. Further inquiries can be directed to the corresponding authors.
